# Vertebrate DNA in Fecal Samples from Bonobos and Gorillas: Evidence for Meat Consumption or Artefact?

**DOI:** 10.1371/journal.pone.0009419

**Published:** 2010-02-25

**Authors:** Michael Hofreiter, Eva Kreuz, Jonas Eriksson, Grit Schubert, Gottfried Hohmann

**Affiliations:** 1 Research Group Molecular Ecology, Max Planck Institute for Evolutionary Anthropology, Leipzig, Germany; 2 Department of Biology, University of York, York, United Kingdom; 3 Department of Primatology, Max Planck Institute for Evolutionary Anthropology, Leipzig, Germany; 4 Animal Ecology, Evolutionary Biology Centre (EBC), Uppsala University, Uppsala, Sweden; Georgia State University, United States of America

## Abstract

**Background:**

Deciphering the behavioral repertoire of great apes is a challenge for several reasons. First, due to their elusive behavior in dense forest environments, great ape populations are often difficult to observe. Second, members of the genus *Pan* are known to display a great variety in their behavioral repertoire; thus, observations from one population are not necessarily representative for other populations. For example, bonobos (*Pan paniscus*) are generally believed to consume almost no vertebrate prey. However, recent observations show that at least some bonobo populations may consume vertebrate prey more commonly than previously believed. We investigated the extent of their meat consumption using PCR amplification of vertebrate mitochondrial DNA (mtDNA) segments from DNA extracted from bonobo feces. As a control we also attempted PCR amplifications from gorilla feces, a species assumed to be strictly herbivorous.

**Principal Findings:**

We found evidence for consumption of a variety of mammalian species in about 16% of the samples investigated. Moreover, 40% of the positive DNA amplifications originated from arboreal monkeys. However, we also found duiker and monkey mtDNA in the gorilla feces, albeit in somewhat lower percentages. Notably, the DNA sequences isolated from the two ape species fit best to the species living in the respective regions. This result suggests that the sequences are of regional origin and do not represent laboratory contaminants.

**Conclusions:**

Our results allow at least three possible and mutually not exclusive conclusions. First, all results may represent contamination of the feces by vertebrate DNA from the local environment. Thus, studies investigating a species' diet from feces DNA may be unreliable due to the low copy number of DNA originating from diet items. Second, there is some inherent difference between the bonobo and gorilla feces, with only the later ones being contaminated. Third, similar to bonobos, for which the consumption of monkeys has only recently been documented, the gorilla population investigated (for which very little observational data are as yet available) may occasionally consume small vertebrates. Although the last explanation is speculative, it should not be discarded a-priori given that observational studies continue to unravel new behaviors in great ape species.

## Introduction

Despite being as closely related to humans as are chimpanzees (*Pan troglodytes*), bonobo (*Pan paniscus*) behavior appears to deviate from that of chimpanzees and humans. This difference is most obvious when looking at dominance relationships between males and females [1]. In chimpanzees and most human societies, adult males dominate females and have priority of access to food sources. In addition to exhibiting physical and social dominance, males cooperate in a number of behaviors, including patrolling the territory and hunting of mammalian prey [2], [3], [4], [5]. In contrast, while sexual dimorphism in body and canine size in bonobos is similar to chimpanzees, male and female bonobos are co-dominant and males do not cooperate [6]. Behavioral observations suggest that females have priority of access to food sources and commonly share food among each other excluding the males [7], [8], which could reflect both male deference and female-female cooperation [9], [10].

Another behavior that is often cited as being different between the two *Pan* species is the frequency of hunting and the selection of prey species [11]. Unlike chimpanzees, which almost exclusively hunt a single species of arboreal primate, the red colobus monkey (*Colobus badius*) [12], bonobos are reported to only occasionally hunt and eat small mammals such as rodents and forest antelopes [13], [14]. However, the majority of information on bonobos comes from two habituated communities situated in the same geographical area and therefore, may not be representative for the species. As comparative approaches across many study sites have demonstrated significant differences in behavior among different chimpanzee communities [15], [16], [17], [18], the few habituated bonobo communities are unlikely to represent the full spectrum of bonobo behavior. Furthermore, direct observations on hunting and meat consumption depend largely on the state of habituation and even when subjects are very tolerant of human observers, consumption of small prey may not always be seen.

Behavioral observations at the study site of Lui Kotale, Salonga National Park, Democratic Republic of Congo [19] provided evidence for the consumption of vertebrate meat by bonobos. Macroscopic analyses of fresh feces yielded samples of hair, bone and cartilage providing indirect evidence for meat consumption. Together with records from direct observations, this information suggested that bonobos at Lui Kotale may consume meat more often than bonobos at other sites. In addition, field work at Lui Kotale has furnished the first cases of hunting and consumption of diurnal, group living primates such as red-tail monkey, Wolf's guenon and black mangabey by bonobos [20], [21]. To examine whether meat consumption by bonobos does occur more frequently than previously reported, we analyzed a large number of feces collected over a period of 20 months from non-habituated bonobos at Lui Kotale and surrounding areas for traces of mitochondrial DNA (mtDNA) from other vertebrate species. This approach allowed us to screen for a wide range of potential prey species including rare cases that might be missed by direct observations because of their small size or because they are consumed infrequently.

As DNA from potential prey species is usually degraded to a substantial degree in predator feces [22], we implemented several of the measures for work with ancient DNA to avoid contamination [23]. However, apart from contamination occurring during processing of samples in the laboratory, there are two additional sources of contamination. First, contamination of chemicals, which has recently been shown to potentially play a role not only with regard to contamination with human DNA but also with DNA from domesticated species like cattle or pig [24] has to be considered. Second, samples themselves may be contaminated with DNA of various sources, potentially even before they are collected [25], [26]. To control for these potential problems, we also amplified DNA from 78 gorilla feces, assuming that samples of this species, which is considered to refrain from consumption of vertebrate meat, do not contain DNA from vertebrate species.

## Results

**Figure 1:**
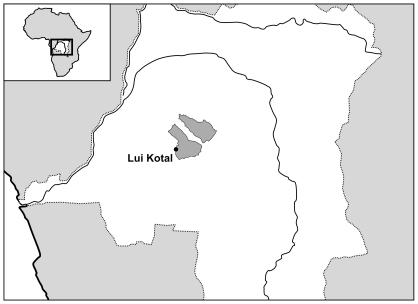
The study site of Lui Kotale is located on the western border of Salonga National Park (shaded area) in the center of the Congo basin, Democratic Republic of Congo.

**Figure 2:**
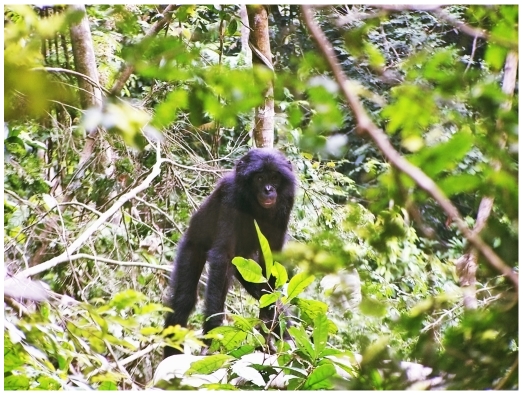
Juvenile bonobo in the natural environment at Lui Kotale.

The study area is situated in the South of the Congo River basin, Democratic Republic of Congo and includes the region of Lui Kotale and adjacent forest areas (Fig. 1 and 2). Samples consisted of 128 feces samples that were collected by two of the authors (JE and GH) between April 2002 and December 2003. We considered only samples that could be unambiguously assigned to individual nests and collected the feces in the early morning immediately after the bonobos left the nest site. Thus, each sample from a particular date should represent a different individual. All samples were screened for the presence of mammalian, bird and lizard DNA using twelve different primer pairs (Text S1). Amplification primers and conditions were designed to preclude amplification of bonobo mtDNA.

**Figure 3:**
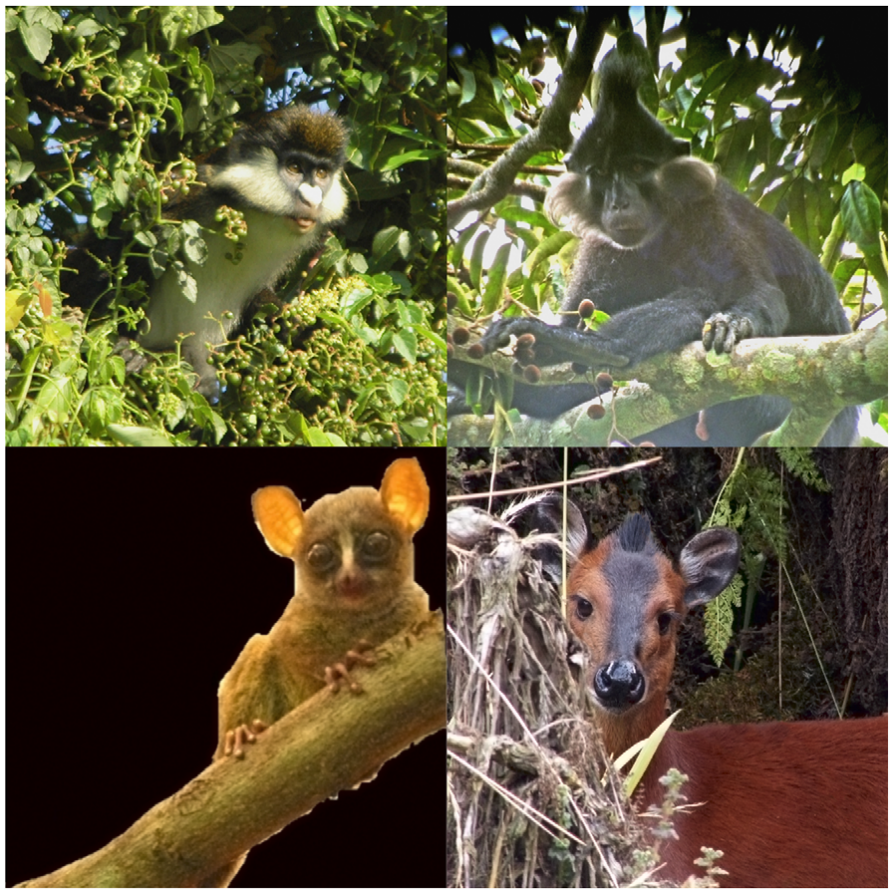
Four of the mammalian species that were identified from bonobo feces and shown by observational studies to represent bonobo prey. Top left: red-tailed monkey; top right: crested mangabey; bottom left: dwarf Galago; bottom right: duiker.

Separation and visualization of the PCR products using gel electrophoresis and ethidium bromide staining revealed that of the 3432 PCRs performed on samples, 115 produced products of approximately the expected lengths. Those products were sequenced and compared to published sequences in GenBank via BlastSearch [27]. In many cases the sequence length obtained after trimming the primers deviated from the expected fragment length, but to a degree below the resolution of standard agarose gels. Nevertheless, these sequences were also included in further analyses. The best matches in GenBank included mtDNA sequences from two monkey species, two rodent species, a galago species, at least one duiker species, pig, domestic dog, cat, and cattle, human nuclear DNA sequences, bonobo mtDNA sequences, and DNA sequences from one species of bacteria and two sequences tentatively assigned to water chevrotain and a bird species, respectively (Fig. 3; see Text S1, Fig. S1 and Table S1 for details). The amplification of bonobo mtDNA in two cases shows that amplification of bonobo DNA was not precluded by all primer pairs. Several products showed only similarities over short lengths (below 25 bp) or with less than 95% identity to any sequence in GenBank, as did 16 products of approximately correct length obtained from 1104 PCR and extraction negative controls. Due to the short length of the amplification products, species from mammalian families different from the target groups sometimes also had very similar Blast hits. However, these were always poorer matches. Moreover, while the best matching non-domestic species occur in the sampling region, the species from mammalian families different from the target groups showing close matches by BlastSearch can be excluded on biogeographical grounds (Text S1 and Table S1).

To control for possible contamination, we also used the three primer pairs that most frequently yielded results for bonobo feces (duiker, monkey and pig) on a sample of 78 gorilla feces. Gorillas eat insects [28] but, to our knowledge, have never been observed to consume vertebrates [29], even though they are known to consume meat in zoos when given the opportunity [30]. As no gorillas occur in the Lui Kotale region, we used gorilla feces from Loango National Park (Gabon). While we did not obtain any positive results for the pig primers, five samples showed positive results for the monkey primer pair (three in duplicates, two in only one of the two attempts for each sample), and three samples also for the duiker primer pair (one in duplicate, two in only one attempt). Interestingly, the sequences were all different from those obtained for the bonobo feces and the closest matches fitted to species that occur in Loango rather than to species from Lui Kotale.

## Discussion

### Species Detected

The sequences of mammalian origin obtained from the bonobo feces can be divided into three categories: first, sequences that are most likely of local origin and whose DNA therefore most likely originates from the feces (Table 1); second, sequences that most likely represent contamination of either the samples during handling or of the reagents used during extraction and amplification; and third, the pig sequences, which could belong to either category, as the fragment amplified does not allow distinction of domestic pigs from the local wild hogs. For the remaining sequences, the distinction is based on the fact that six (seven if water chevrotain is included) of the identified species not only occur in the region from which the bonobo samples originate, but are also plausible as prey species (in terms of size) while three of the species (domestic dog, domestic cattle, and domestic cat) are not plausible as bonobo prey. As noted, the situation is less clear for the pig sequences. However, red river hogs (*Potamochoerus porcus*) are common at LuiKotale and recently, the consumption of piglets has been observed (A. Fowler personal communication). Moreover, our failure to amplify pig DNA from the gorilla feces indicates that reagent contamination [24] is an unlikely explanation for the observed results and suggests that the pig DNA may indeed have been endogenous to the bonobo feces. The sequence obtained using bird primers, although undoubtedly from a bird species (see Text S1 and Table S1) was too distant (62/69 bp identity) to any sequence in GenBank to allow species identification. As it is not closely related to any domestic bird species (such as chicken or turkey), it most likely represents DNA endogenous to the analyzed feces rather than contamination from chemicals [24] or laboratory handling. Similarly, the identification of the water chevrotain sequence is tentative for several reasons, although this species occurs in the region of Lui Kotale (see Text S1 and Table S1).

**Table 1:** Samples that yielded putative prey DNA.

Primer	Sample nr.	Collection date	Nest group nr.	Species detected
**monkey**	170	13.09.02	6	Cercopithecus ascanius
	171	13.09.02	6	Cercopithecus ascanius
	172	13.09.02	6	Cercopithecus ascanius
	250	21.11.02	13	Cercopithecus ascanius
	254	24.11.02	14	Cercopithecus aethiops
	320	19.01.03	18	Cercopithecus ascanius
	442	07.04.03	24	Cercocebus aterrimus
	443	07.04.03	24	Cercocebus aterrimus
	444	07.04.03	24	Cercocebus aterrimus
	447	07.04.03	24	Cercocebus aterrimus
**rodent**	180	19.09.02	8	Anomalurus sp.
	181	19.09.02	8	Anomalurus sp.
	319-1	19.01.03	18	Protoxerus stangeri
**duiker**	203	03.10.02	9	Cephalophus spadix
	315	19.01.03	18	Cephalophus natalensis
	316	19.01.03	18	Cephalophus spadix
	320	19.01.03	18	Cephalophus spadix
	321	19.01.03	18	Cephalophus spadix
	379	15.02.03	20	Cephalophus spadix
	380	15.02.03	20	Cephalophus spadix
	381	15.02.03	20	Cephalophus spadix
	442	07.04.03	24	Cephalophus spadix
**galago**	92	05.07.02	4	Galago senegalensis
**bird**	183	19.09.02	8	unidentified bird
**tragulus**	33	20.05.02	2	Hyemoschus aquaticus

Primer indicates the primer pair that was used to amplify DNA from the respective feces; sample numbers were given chronological during the sampling period. Each number represents a unique sample. The “Species detected” are those that are most likely when combining the results of the BlastSearch and data on the occurrence of species at Lui Kotal. Pig sequences were not included as they could be derived from contamination of chemicals.

The common livestock in the villages around the park are chicken, sheep and goat while cattle and cats are completely absent. Villagers keep dogs and these may enter the forest when people move to temporary fishing camps. However, unlike in other regions in the Congo basin, local hunters in the villages adjacent to the study site do not use dogs for hunting and we have no positive evidence that dogs have crossed the Lokoro River separating the study site from community forests during the period of data collection. Hence, circumstantial evidence suggests that sequences from domestic animals are contaminations rather than traces of mammalian prey. Exclusion of the dog, cattle, and cat sequences from further consideration is also supported by the fact that similar contamination of PCR results with DNA from domestic animals has been reported before and attributed to either handling of the samples or contamination of chemicals [24], [25]. Finally, these sequences were only found in five samples representing five non-replicable sequences.

Even if one accepts that these five sequences represent laboratory or reagent contamination, as previously reported [24], these results argue for extremely careful interpretation of results from molecular analyses using feces DNA. The problems surrounding such studies are further emphasized when the remaining results of the bonobos are analyzed, consisting of 41 positive amplifications from 23 feces with best matches to species living in the region of Lui Kotal, and 19 positive amplifications from 16 feces matching pig sequences for which we cannot determine if they originate from the wild species or domestic pigs. In itself these results may be taken as evidence for frequent meat consumption by this bonobo population. However, the detection of DNA from domestic animal species that are absent in the forest generally calls for caution when interpreting results of genetic approaches to studies on feeding behavior. The presence of DNA from wild mammals in fecal samples from gorillas further complicates the interpretation of our results. While we did not detect sequences from pigs or domestic livestock, monkey and duiker sequences were obtained at frequencies similar to the bonobo feces (5/78 [6%] vs. 10/128 [8%] and 3/78 [4%] vs. 9/128 [7%], respectively, totaling 15% for bonobos versus 10% for gorillas). We suggest several possible and mutually non-exclusive explanations for the results.

### Origin of the Sequences

#### Contamination

The first and most simple explanation is that, like the cat, dog and cattle sequences, the remaining sequences detected may represent contamination of sample material. Contamination of the chemicals used in the analyses with duiker or monkey sequences, as it has been shown possible for DNA of domesticated species [24], [25] is highly unlikely. Given that the sequences obtained from the gorilla samples are different to all sequences obtained previously from the bonobos and were never handled before in our laboratory, we feel confident to also rule out contamination during handling of samples in the laboratory. Thus, the most likely explanation is that samples were contaminated in the forest during or prior to collection. Support for this explanation comes from the fact that the sequences from monkeys and duikers detected in the samples from bonobos and gorillas, respectively, matched very well with faunal assemblies at Lui Kotale (Congo) and Loango (Gabon), respectively. This type of contamination, occurring before sampling, is most problematic as it is impossible to control for [26]. While this explanation is in line with the assumption of the accepted, strictly herbivorous, diet of gorillas, it is difficult to reconcile with the results obtained from bonobo feces. First, there is direct evidence for the consumption of meat from duikers, rodents, galago [31], red river hogs, and as recently reported, also diurnal group living monkeys [20], [21]. Moreover, the monkey species that were directly observed to be hunted and consumed by bonobos are the same species we identified using our molecular approach. Second, the size of the species of wild mammals detected by genetic markers fits the size of animals that can be captured and handled by bonobos, an observation that interestingly also applies to the findings from the gorilla feces. Sequences from large mammals such as forest buffalo and leopard were not detected. Likewise, sequences from the golden bellied mangabey, a relatively large, terrestrial primate did also not appear. Finally, samples were picked up shortly after the bonobos left their nest sites, and specimens of the putative prey items were handled neither in the camp nor in the laboratory. Taken together, while the detection of DNA of vertebrates in fecal samples of bonobos match observational data from the same population we can not disregard contamination as an explanation for some or even the majority of the results. However, two alternative explanations warrant consideration.

#### Differences in sampling scheme

Feces from gorillas included samples that were several days old while all bonobo samples were fresh. Therefore, the results obtained from gorillas are more likely to reflect contamination of samples prior to collection, while the bonobo samples are less likely to have become contaminated prior to sampling. Given that samples from bonobos but not from gorillas contained sequences from domestic animals, the sampling scheme alone does not help to tackle the origin of all the sequences obtained from the feces. In other words, studies using molecular methods to detect DNA of diet items in feces might be highly prone to artefacts, especially when dealing with rare diet items that would be expected to be found infrequently. If defecation is observed and samples are collected immediately, the likelihood of contamination should be reduced. However, given the possibility of a detectable number of false positives resulting from environmental contamination we think studies investigating this issue are urgently warranted, especially as this potential source of contamination could easily be mistaken as endogenous DNA.

#### Meat consumption

Until recently, hunting of diurnal, group living primates by bonobos was considered to be absent [32] and detection of DNA from such species in bonobo feces would have certainly been considered to be contamination. From observations at Lui Kotale it is known that bonobos of this population hunt and consume the meat of several primate species. Given the paucity of information on the behavior and food selection of gorillas at Loango, we think that the possibility exists that the results from gorilla feces originate from endogenous DNA that has passed the digestive tract. There are various ways to explain this. First, gorillas, in contrast to bonobos, eat highly carnivorous driver ants that scavenge on carcasses, bones and other animal remains and by doing so could take up DNA from their prey. In this context it should be noted that a detailed morphological analyses of 177 gorilla feces from this region did not yield any evidence, such as hair or bone remains, for vertebrate consumption by gorillas (C. Boesch, personal communication). Second, similar to bonobos, some gorilla populations may feed on other vertebrates, either by hunting or by picking up already dead animals. In captivity, gorillas readily consume meat and other animal foods [30] and there is evidence for cannibalism in wild populations [33]. We admit that any suggestion of gorillas consuming vertebrate meat is highly speculative. However, given that Loango gorillas are not yet habituated, the molecular data remain to be tested with direct observations. Our molecular study on bonobos was completed before the first observation of bonobos hunting and consuming both monkeys and piglets and in this way, the results obtained by molecular techniques preceded behavioral observations. Therefore, we think it would be a grave mistake–and indeed non-scientific reasoning–to disregard the molecular results based only on the fact that there is so far no observational evidence available for a certain behavior. We do not claim that our results are proof for the consumption of vertebrate meat by gorillas, but we would like to point out that it is one possible explanation that can only be discarded after direct observations become possible.

### Conclusions

Our results emphasize both the potential and the weakness of molecular diet analyses using DNA from feces. For bonobos, the findings obtained by the molecular approach preceded direct evidence from behavioral observations. This suggests that molecular studies have the potential to be inductive by drawing the attention of researchers to new topics. However, the presence of DNA from domestic animals in fecal samples from bonobos and the fact that we also found monkey and duiker DNA in feces from gorillas suggests that results obtained exclusively by molecular studies may be prone to misinterpretation due to contamination. The detection of DNA from monkeys and duikers in gorilla feces from Loango invites speculations concerning the food habits of this population and is a challenge for future field studies. Further studies investigating the reliability of DNA sequence data from feces and the development of methods to distinguish truly endogenous DNA from environmental contamination are necessary before such analyses can be used as sole evidence for novel behavior. In the meantime, molecular feces analyses are important for directing the attention of scientists to unusual aspects of feeding behavior–for example for possible meat consumption of gorillas at Loango.

## Materials and Methods

Fecal samples (N = 128) from bonobos (*P. paniscus*) from the region of Lui Kotale, Salonga National Park, Congo basin, Democratic Republic of Congo were used as DNA source. Permission to export fecal samples was granted by the Institut Congolais pour la Conservation de la Nature (ICCN). All samples were collected between April 2002 and December 2003 and consisted of approximately 5 g portions of fresh feces transferred directly onto silica (68 samples) or suspended in RNA-later® (Ambion) (60 samples) [34] and stored at 4°C until processing.

Samples were extracted using the QIAamp DNA stool kit following the protocol provided by the supplier with some changes [34]. DNA extracts were tested for DNA content using a quantitative PCR (ABI 7700) system targeting a 81 bp (including primers) fragment of the nuclear c-myc gene following the protocol from Morin et al. [35] except that 16 ug BSA were used per reaction. Bonobo samples that showed very low DNA contents (below 25 pg/2 ul) were extracted a second time. For all further experiments we used both extracts. Thus for 14 samples we used DNA from two independent extractions and for one from three extractions, while the remaining 113 samples were extracted only once. Feces samples (N = 78) for gorillas from Loango National Park, Gabon, were sampled and extracted as described for the bonobos. DNA was kindly supplied by Mimi Arandjelovic.

To determine DNA from possible prey items we designed twelve primer pairs, each specific for amplification of mtDNA from phylogenetically closely related groups of animal species living in the habitat of the Lui Kotale bonobo population and, based on size and other biological features, representing potential prey items (see Text S1 for details on species selection and Table S1 for primer sequences and expected length of the amplicons). All primer pairs were designed to exclude amplification of bonobo mtDNA due to mismatches at the 3′-end of at least one primer [36]. Prior to use on the feces DNA all primer pairs were tested on DNA from representative species and PCR conditions optimized with regard to annealing temperature in order to obtain maximal sensitivity. For amplification of prey DNA we used 2 µl extracted DNA in reactions consisting of 1x PCR buffer II (Applied Biosystems), 2.5 mM MgCl_2_, 0.25 µM each primer, 0.25 µM each dNTP (Amersham Biosciences), 0.5 U AmpliTaq Gold (Applied Biosystems) and 16 ug BSA in a final volume of 20 µl. For each sample, PCRs were performed in duplicate on independent plates to avoid cross-contamination. Throughout all experiments we performed extraction and PCR negative controls alongside with the feces extractions to monitor for possible contamination. To make sure that failure to amplify DNA from a certain species group from the feces is not due either to general PCR failure or low sensitivity, we included DNA at low concentration from representative species as positive control in all amplifications. In cases when amplification of the positive controls failed we repeated the amplification for all samples.

Amplifications were performed on a PTC-225 Thermo cycler (Biozym) using a 3-min initialization step at 94°C followed by 50 cycles consisting of 30 sec at 93°C, 45 sec at 50°C–62°C (depending on the primer pair used) and 45 sec at 72°C and a final elongation step of 10 min at 72°C. The high number of 50 PCR cycles was used due to the likely low quantities of prey DNA in feces [22], [37]. PCR products were visualized on 2.5% Seakem®LE-agarose-gels (BMA) stained with ethidium bromide. All amplifications of expected size were cloned using the TOPO® TA cloning kit (Invitrogen). Products from reactions showing primer dimers or secondary bands were isolated from the gel and purified using the QIAquick gel extraction kit according to the manufacturer's instructions prior to cloning.

Single colonies were isolated from agar plates for colony PCRs [38] using M13 universal primers. Colony PCR products were purified using the BioRobot 9600 system (QIAgen). Cycle sequencing was performed as described previously and from each primary amplification at least eight clones were sequenced on an ABI3730 DNA analyzer. All sequences were analysed using the program package SeqMan (Applied Biosystems) and compared to the sequences available in GenBank using the program BlastSearch ([27]; see also Text S1).

## Supporting Information

Text S1 Supporting information text
(0.04 MB DOC)

Table S1 Results of BlastSearch using all non-bacterial sequences that yielded identities larger than 90% over a length >25 bp. The columns give the primer pair used for amplification, the sample number, the output order of the Blast result, the scientific species name, its classification in the taxonomic system, the genomic region to which the fragments match, the fragment length after removal of the primer sequences, number and percentage of identities and gaps, respectively, and information on the occurrence of each species in the region of LuiKotal. Cases when species with identical Blast matches have different geographical distributions are indicated by corresponding colors in the columns for “species” and “occurrence in the sampling region”, respectively.
(1.33 MB TIF)

Figure S1 Sequence alignment of all clones representing putative prey sequences. In cases where we obtained duplicate sequences, clones from both amplifications are shown. The numbers before the sequences show the feces tube number, the extraction number, PCR number and clone number. We aligned the sequences to the closest match from GenBank except for the putative water chevrotain sequence, for which we used the sequence obtained from bonobo feces as reference. Dots indicate identity to the reference sequence; differences are shown by the respective nucleotide symbol or a dash in case of indels. a) duiker sequences obtained using duiker primers; b) duiker sequences obtained using rodent primers; c) Cercopithecus sequences obtained using monkey primers; d) Cercocebus sequences obtained using monkey primers; e) rodent sequences obtained using rodent primers; f) galago sequences obtained using galago primers; g) bird sequences obtained using bird primers; h) putative water chevrotain sequences obtained using Tragulidae primers.
(3.20 MB DOC)
