# Supplementary material for: Vertebrate DNA in Fecal Samples from Bonobos and Gorillas: Evidence for Meat Consumption or Artefact?
Source: PLoS One. 2010 Feb 25;5(2):e9419. doi: 10.1371/journal.pone.0009419 (PMC2828480; doi:10.1371/journal.pone.0009419)
Supplement: Text S1 — Supporting information text (0.04 MB DOC) [file pone.0009419.s001.doc]

**Supporting information**

We designed primer pairs for specific amplifications of short fragments of mtDNA from twelve species groups. Primers were designed based on sequences publicly available from GenBank. The species groups targeted were monkeys, duiker, galago, rodents, bats (Megachiroptera), Tragulidae (water chevrotain), hyrax, cats, pigs, African shrews, birds and lizards. Whenever possible, primer design was based on sequences from species occurring in the Congo basin region and/or close relatives. However, in several cases, no sequences of the respective species or close relatives were available in GenBank. In those cases, such as the Tragulidae, for which only sequences from the Asian representative of the family were available, we used the next closest relative in addition to a few more distant species. In case of the bird and lizard primers we used very general primers targeting these groups, but at the same time, as in all cases, excluding the amplification of bonobo mtDNA due to a 3’ terminal mismatch to the respective bonobo sequence [1]. Due to these restrictions it is very probable that the DNA from a number of potential prey species is amplified only with low sensitivity or not at all using these primers.

Altogether, 208 of the 3432 PCRs performed on the 128 samples resulted in amplification products. However, a number of amplification products deviated from the target length of the respective amplification products, indicating unspecific amplification of other DNA sources in the extracts. This phenomenon has been observed before [2] and is expected to occasionally occur if large numbers of amplifications from complex DNA mixtures such as feces extracts are performed. Of the 131 amplification products showing no visible length difference to the respective PCR fragments (+/- 20 bp upon sequencing) on agarose gels, upon sequencing 57 were found to be of either bacterial or unknown origin. The remaining sequences match with high similarity (96-100%) to human nuclear DNA and mtDNA sequences from bonobo, pig, dog, cattle, cat, two monkey species, two rodent species, duiker, galago and a bird species (Table S1). For the sequence obtained using the tragulus primers, the closest match in GenBank was to a mole species with only 92% identity. However, for the African genus from the family Tragulidae, the water chevrotain (*Hyemoschus*), no sequences are available in GenBank. Therefore we sequenced the corresponding DNA fragments from a museum specimen kindly provided by the Museum für Naturkunde, Berlin and a feces sample from Lui Kotal. However, both samples revealed multiple sequences in several amplifications. Interestingly, all these sequences were similar to each other, with one sequence being identical to that obtained from the bonobo feces sample no. 33. A phylogenetic analysis using all obtained haplotypes together with several Ruminantia species obtained from GenBank showed all putative water chevrotain sequences to be monophyletic and most closely related to the Asian member of the family Tragulidae, the genus *Tragulus* (results not shown). This result suggests that the sequences obtained from the water chevrotain feces, museum specimen and the bonobo feces indeed originate from water chevrotain DNA. The fact that multiple sequences were recovered could be explained by heteroplasmy, the occurrence of nuclear copies of the mitochondrial DNA, and in the case of the museum specimen also cross-contamination with other samples from the same species. Due to these uncertainties, the identification of water chevrotain as bonobo prey is tentative and further work will be necessary to show whether this species indeed belongs to the prey spectrum of bonobos at Lui Kotal – as shown by behavioral observations for arboreal monkeys and red river hogs – or not.

When considering Table S1, it has to be taken into account that Blast provides a local alignment, i.e. in cases where the query sequence differs to GenBank sequences at either end of the query sequence, the alignment output given by Blast will be shorter than the query sequence. For example, the bird sequence has a Blast output of 53/57 bp identity. However, the complete query is 69 bp in length, and manual comparison of the query sequence to the closest match in GenBank reveals 62/69 bp identity. We also found three different sequences when amplifying from sample 320 using the rodent primers (Table S1 and Fig. S1b). Two of these likely represent two different duiker specimens, while for the third one it is not possible to positively assign it to a species, although the Blast results hint to a small gazelle species. Thus, it is possible that the feces from individual 320 contained DNA from four different species, as it was also positive for monkey DNA. However, as we could not positively identify the putative gazelle species and cannot exclude that the two duiker sequences are due to heteroplasmy of one individual or result from DNA damage, we did not consider the two additional sequences in our calculations. We would also like to note that contamination seems an exceedingly unlikely explanation for this result. Given the comparatively low overall success rate we consider it very unlikely that we would have contaminated a single sample with four different sequences that were never handled in the laboratory. However, the chance that a sample got contaminated in the field with DNA from four different species during the short time span from the bonobos leaving their nests to sampling seems even more remote.

With regard to species identification, it also needs to be taken into account that intra-species polymorphisms may exist and not all species from the region have been sequenced, a problem highlighted by the identification problems of the water chevrotain sequence. However, it is noteworthy, that the species or genera showing the closest match by BlastSearch all occur in the sampling area, giving strong support for the identifications. In contrast, this is often not the case for the less good matches (Table S1).

**Supporting references**

1. Kwok S, Kellogg DE, McKinney N, Spasic D, Goda L, et al. (1990) Effects of primer-template mismatches on the polymerase chain reaction: human immunodeficiency virus type 1 model studies. Nucleic Acids Res 18: 999-1005.

2. Bradley BJ, Vigilant L (2002) False alleles derived from microbial DNA pose a potential source of error in microsatellite genotyping of DNA from faeces. Molecular Ecology Notes 2: 602-605.
