# Supplementary figures and images for: Vertebrate DNA in Fecal Samples from Bonobos and Gorillas: Evidence for Meat Consumption or Artefact?
Source: PLoS One. 2010 Feb 25;5(2):e9419. doi: 10.1371/journal.pone.0009419 (PMC2828480; doi:10.1371/journal.pone.0009419)

a)


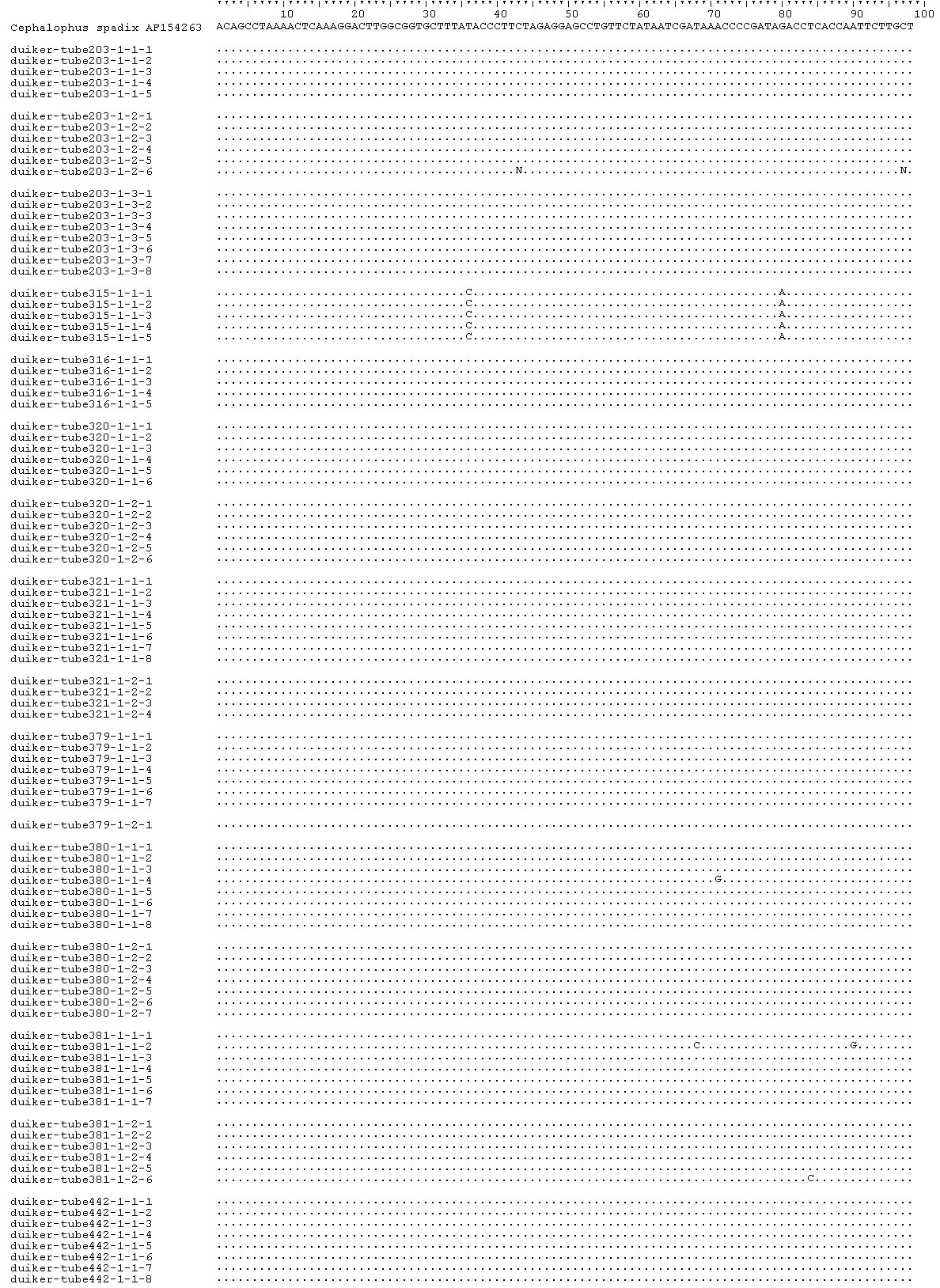


b)


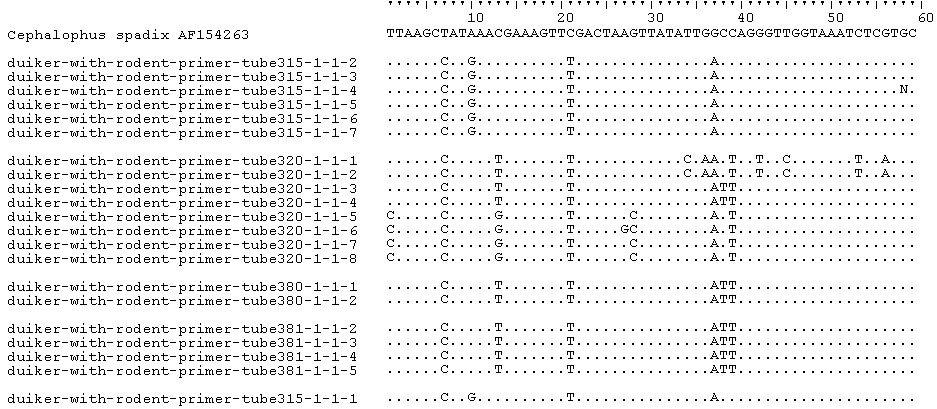


c)


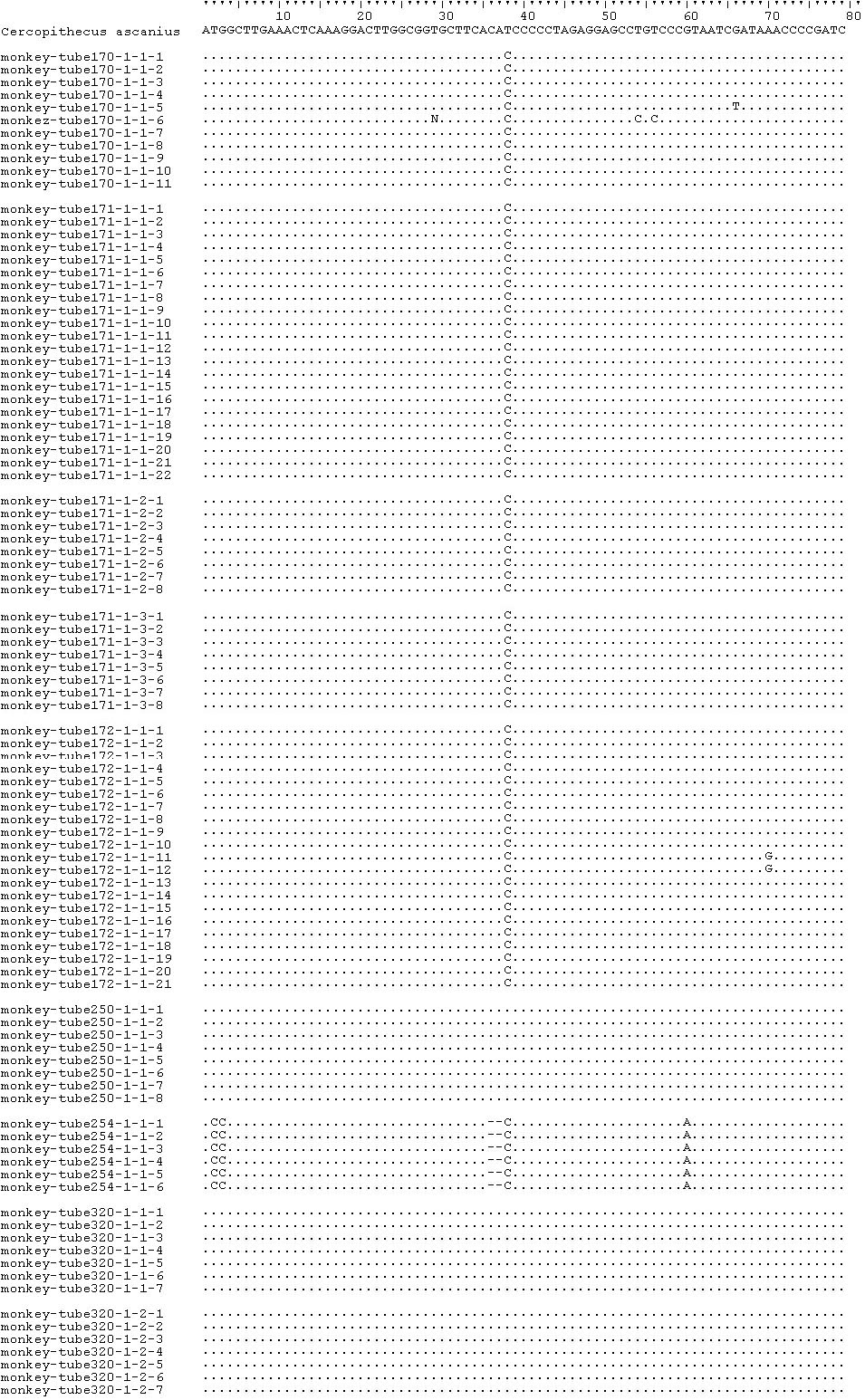


d)


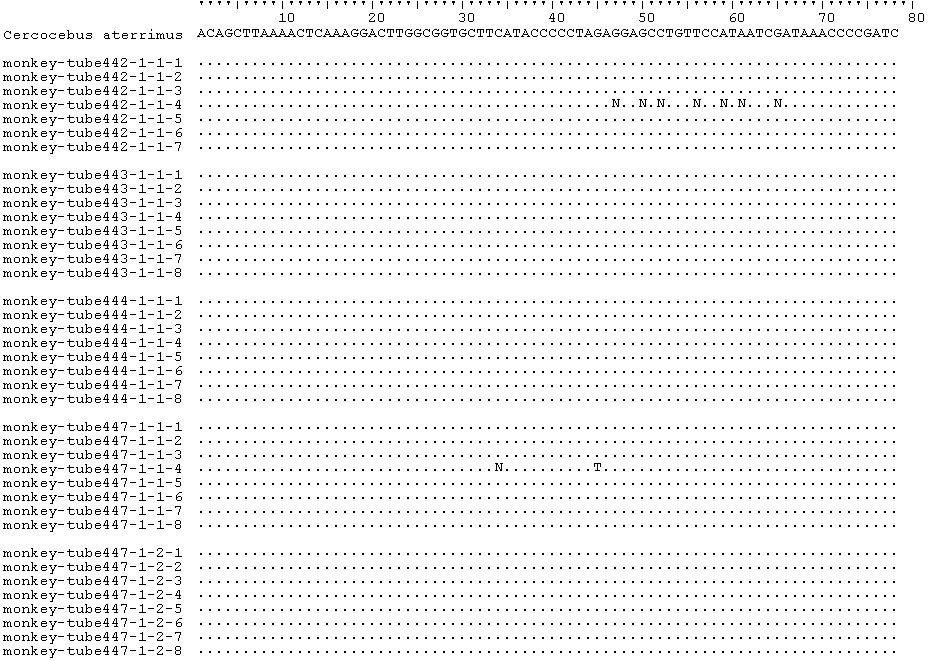


e)


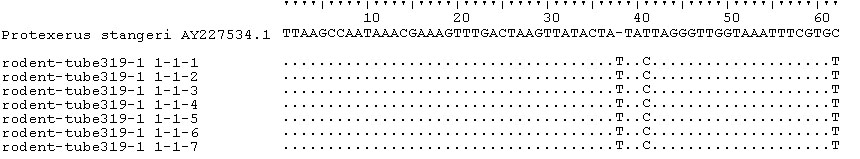


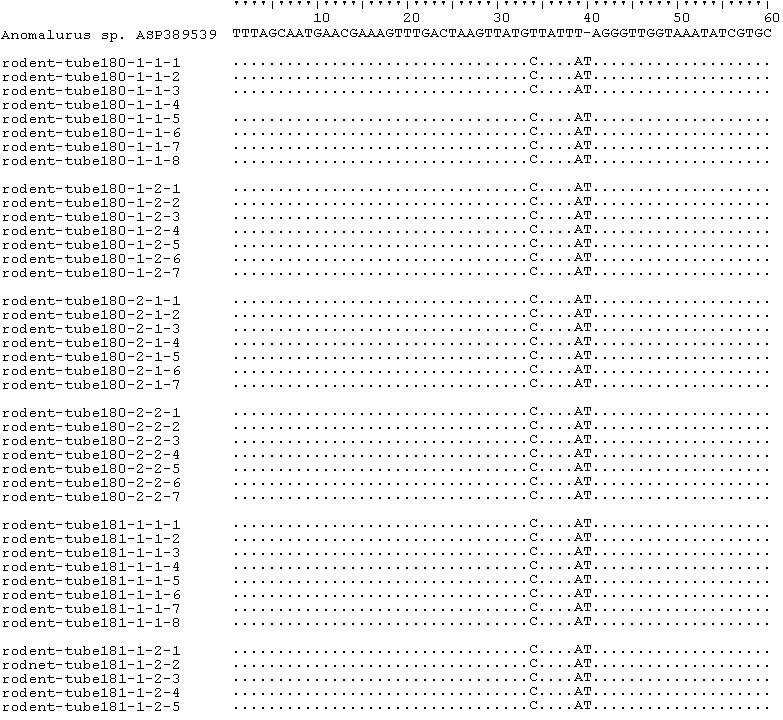


f)


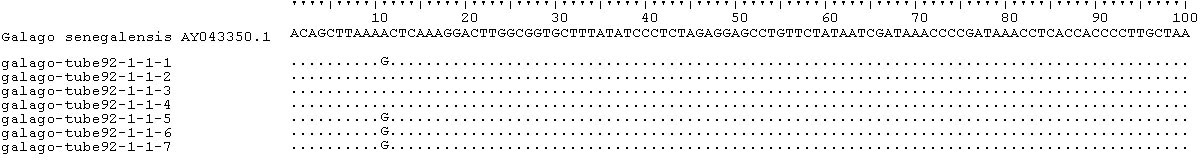


g)


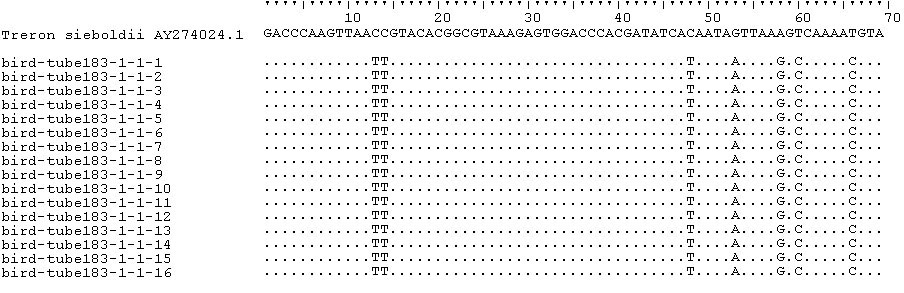


h)


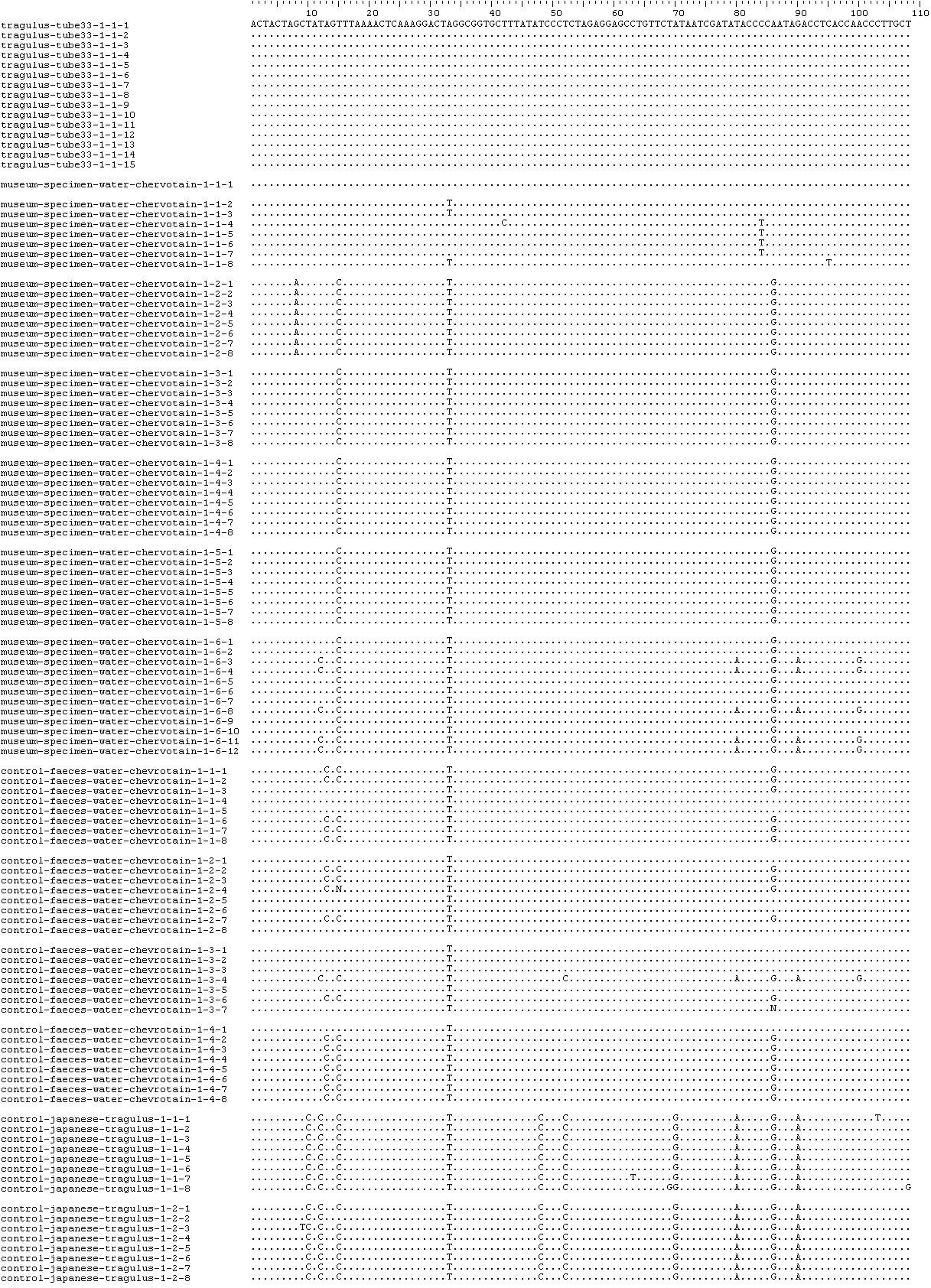

Supplement: Figure S1 — Sequence alignment of all clones representing putative prey sequences. In cases where we obtained duplicate sequences, clones from both amplifications are shown. The numbers before the sequences show the feces tube number, the extraction number, PCR number and clone number. We aligned the sequences to the closest match from GenBank except for the putative water chevrotain sequence, for which we used the sequence obtained from bonobo feces as reference. Dots indicate identity to the reference sequence; differences are shown by the respective nucleotide symbol or a dash in case of indels. a) duiker sequences obtained using duiker primers; b) duiker sequences obtained using rodent primers; c) Cercopithecus sequences obtained using monkey primers; d) Cercocebus sequences obtained using monkey primers; e) rodent sequences obtained using rodent primers; f) galago sequences obtained using galago primers; g) bird sequences obtained using bird primers; h) putative water chevrotain sequences obtained using Tragulidae primers. (3.20 MB DOC) [file pone.0009419.s003.doc]
